# Effect of* Helicobacter pylori* Infection on Serum Lipid Profile

**DOI:** 10.1155/2018/6734809

**Published:** 2018-06-03

**Authors:** Mohamadreza Haeri, Mahmoud Parham, Neda Habibi, Jamshid Vafaeimanesh

**Affiliations:** ^1^Department of Biochemistry, Qom University of Medical Sciences, Qom, Iran; ^2^Clinical Research Development Center, Qom University of Medical Sciences, Qom, Iran; ^3^Gastroenterology and Hepatology Disease Research Center, Qom University of Medical Sciences, Qom, Iran; ^4^Gastrointestinal and Liver Diseases Research Center, Iran University of Medical Sciences, Tehran, Iran

## Abstract

**Background:**

Some studies suggest a significant relationship between* Helicobacter pylori* infection and atherogenesis; but the mechanism of the relationship is almost unknown. The current study aimed at evaluating the relationship between* H. pylori* infection and serum lipid profile.

**Patients and Methods:**

The current study was conducted on 2573 patients, from 2008 to 2015. The serum anti-*Helicobacter pylori* antibody titer and serum lipid profile were assessed in the study population; data were statistically analyzed by SPSS version 16. *P* values < 0.05 were considered significant.

**Results:**

In the current study, 66.5% of the cases were serologically positive for* H. pylori*. Among male cases, the level of low density lipoprotein (LDL) was higher in patients with* H. pylori* infection, compared with that of the ones without the infection (*P* = 0.03); although level of triglyceride (TG) was higher and the level of high density lipoprotein (HDL) was lower in the cases with* H. pylori* infection; there was no statistically significant difference between the cases with and without* H. pylori* infection regarding the level of HDL and TG. Among female cases, the level of TG was significantly lower in patients with* H. pylori* infection, compared with that of the ones without the infection (*P* = 0.001); but there was no significant difference between the cases with and without* H. pylori* infection regarding the level of LDL and HDL. The mean fasting blood sugar (FBS) in the cases with* H. pylori* infection was significantly higher than that of the ones without the infection (*P* = 0.04).

**Conclusion:**

According to the results of the current study, the levels of LDL and FBS were high among the male cases with* H. pylori* infection. However, in females with* H. pylori* infection the level of TG was low; hence, it seems that the atherogenicity of* H. pylori* affected the level of blood sugar more.

## 1. Introduction

Coronary artery disease is one of the main causes of mortality in the developed countries. Diabetes, lipid profile disorders, hypertension, and smoking are the common risk factors of atherogenesis, which result in the coronary artery disease [1].

Although knowledge on the relationship between the classical risk factors of coronary artery disease has increased, the mechanisms involved in the mortality of the disease were not explained completely. Therefore, studying the relationship between the disease and other risk factors is of great importance.

Different studies investigated the relationship between ischemic heart disease and chronic infection. Some studies reported* H. pylori* infection as a risk factor for coronary artery disease [2, 3].

*Helicobacter pylori* infection is still one of the most common infections worldwide. A significant relationship was reported among* H. pylori* infection, metabolic risk factors, atherogenesis, and cardiovascular diseases in some of the studies [3–5].

It is important to find the mechanism of* H. pylori* pathogenicity in the incidence of cardiovascular diseases. Some of the animal model studies reported some findings in this regard; for example, Elizalde et al. indicated that* H. pylori* infection caused platelet aggregation in rats [6].

Also, Byrne et al. showed that* H. pylori* binds to von Willebrand factor (vWF), which causes platelet aggregation following the interaction with glycoprotein Ib [7].

There are some other reports on the relationship between* H. pylori* infection and chronic progression of atherogenesis; but the mechanism is not identified perfectly yet [2].

Among metabolic risk factors, changes in serum lipid profiles such as low density lipoprotein (LDL) cholesterol and high density lipoprotein (HDL) cholesterol are known as the risk factors for cardiovascular diseases [8].

Different studies showed a significant relationship between the chronic infections and changes in the metabolism of lipids [9, 10]; some other studies also stated that* H. pylori* infection may change the level of serum lipid profile and increase the risk factors of atherogenesis [11].

Can* H. pylori* infection increase the risk of coronary artery diseases by changing serum lipid profile?

Some researchers reported findings contrary to the developed hypothesis [12, 13]. Considering the high prevalence of* H. pylori* infection in Iran [14], investigations on this issue are of great importance; also, because the relationship between lipid profile and* H. pylori* infection is not investigated precisely in the developing countries, the current study was designed, to determine whether Helicobacter pylori infection can be a risk factor for coronary heart disease by affecting the blood lipid profile.

## 2. Materials and Methods

The current study was conducted on 2573 Iranian cases without the history of* H. pylori* eradication therapy, with coronary artery disease, from 2008 to 2015. The age range of cases was 21 to 97 years (44.13 ± 15.48). Totally, 238 cases (98 male and 140 female) were excluded from the study as their serum triglyceride (TG) was > 400 mg/dL; 157 cases (69 male and 88 female) were also excluded from the study due to receiving cholesterol-lowering medication. Finally, results of 2178 cases were analyzed. The current study was approved in the Ethic Committee of Qom University of Medical Sciences, Qom, Iran, and the written consent was obtained from all cases. The case group was infected with* Helicobacter pylori* infection and the control group was non-infected.

### 2.1. Data Collection

To collect data in the current study, a questionnaire was used; the questionnaire included medical history, smoking status, exercising program, menstrual status, and alternative hormone replacement therapy. Smoking status included the continuous, sometimes, and non-smoker options. Antecubital vein blood samples were drained during the morning fasting; serum samples were separated after centrifugation. HDL-cholesterol was measured by a commercial kit including magnesium chloride and phosphotungstate, after sedimentation of lipoproteins including apo B. TG was measured by an enzymatic method. Fasting blood sugar (FBS) was also measured employing the enzymatic method of hexokinase. LDL-cholesterol profile was calculated based on Friedewald formula [15].

C-reactive protein (CPR) was measured by nephelometry technique; amounts were measured to the nearest 0.004 mg/dL. Blood pressure was measured by a trained nurse using a mercury manometer, after 5-minute resting in sitting position. Serum* H. pylori* IgG titer was measured by an enzyme-linked immunosorbent assay (ELISA) kit (Padtan Elm Co., Iran); Ig G titers ≥ 22 U/mL were reported as positive. Statistical analysis was performed by SPSS version 16; *P* values < 0.05 were considered significant.

## 3. Results

The current study was conducted on 2178 cases from 2008 to 2015. Among the cases, 1205 were male and 1132 female. Demographic and clinical data of the cases are shown in Table 1.

According to Table 2, the mean FBS in male cases was significantly higher among the ones with* H. pylori* infection, compared with that of the ones without the infection [105 ± 8 mg/dL versus 101 ± 4 mg/dL] (*P* value = 0.04). The level of LDL was significantly higher among the case with* H. pylori* infection, compared to that of the ones without the infection (*P* value = 0.03); although higher levels of TG and lower levels of HDL were reported among* H. pylori* seropositive cases, there was no significant difference between the infected and non-infected cases regarding the level of TG and HDL (Table 2).

According to Table 3, in female cases, the level of FBS was significantly higher among* H. pylori* seropositive cases, compared with that of seronegative ones (*P* value = 0.04). Also, the level of TG was significantly lower among cases with* H. pylori* infection, compared with the ones without such infection (*P* value = 0.001); but the difference between* H. pylori* seropositive and seronegative cases, regarding the level of LDL and HDL, was insignificant (Table 3).

## 4. Discussion

*Helicobacter pylori* infection is the most common bacterial infection worldwide, especially in the developing countries; its prevalence varies in different countries, 30% in the developed countries versus 80% in the developing countries [16]. The prevalence of* H. pylori* among the current study population was 66.5%.

*Helicobacter pylori* infection may develop many extra-intestinal complications. During the recent years, many studies were conducted on the relationship between* H. pylori *infection and atherosclerotic diseases such as ischemic heart disease [3].

*Helicobacter pylori* may play a role in the development of ischemic heart disease through different methods such as colonization of endothelial cells, changes in lipid profile, hypercoagulation, platelet aggregation, induction of molecular mimicry mechanisms, and progression of low-grade systemic inflammation [17].

The current study aimed at evaluating the relationship between* H. pylori* serological status and serum lipid profile. According to the results of the present study, 66.5% of total population was* H. pylori* seropositive; the prevalence of infection among male and female cases was 68% and 65%, respectively; it was 46.8% and 39.6% in a similar study [18].

According to the results of the current study, in male cases,* H. pylori* seropositivity resulted in significant increase of serum LDL (*P* value = 0.03); although higher levels of TG and lower levels of HDL were observed among* H. pylori* seropositive cases, there was no significant difference between seropositive and seronegative cases regarding the level of HDL and TG. Results of a study conducted in Japan indicated that* H. pylori* infection in Japanese male patients indirectly caused changes in serum lipid profile including an increase in LDL-cholesterol and a decrease in HDL-cholesterol [18]. Also, previously performed studies showed that* H. pylori* infection is associated with lower levels of HDL-cholesterol in Europeans living in the USA [19, 20].

Results of a large epidemiological survey by Laurilaet et al. showed that the level of TG and total cholesterol was significantly higher and the level of HDL-cholesterol was significantly lower in male patients with* H. pylori* infection, compared with the ones without such infection [10]. In a similar study conducted in South Korea, the relationship between* H. pylori* infection and higher levels of LDL-cholesterol was reported [21].

According to the results of the current study, the level of TG was significantly lower in female cases with* H. pylori* infection than the female cases without the infection (*P* value = 0.001); however, the difference between* H. pylori* seropositive and seronegative female cases regarding the level of LDL and HDL was insignificant; the results were consistent with those of a study in Japan [18].

Results of a study showed that in patients with frequent infection with* H. pylori*, the level of LDL and HDL increases and decreases from the base levels, respectively, compared to those of the healthy cases [22]. Although result of a recently conducted study showed that successful eradication of* H. pylori *increases the risk of increased LDL and decreased HDL, it does not reduce the risk of cardiovascular diseases [22].

However, results of the current study and those of some other similar studies to some extent indicated the effect of* H. pylori* on lipid profile in patients with* H. pylori* infection, although results of the current study were not to the extent of justifying the atherogenic effects of* H. pylori*, and it seems that the bacteria induce their possible atherogenic effects through other mechanisms. Results of the current study indicated that the mean FBS in male cases with* H. pylori* infection was significantly higher than that of the ones without the infection [105 ± 8 mg/dL versus 101 ± 4 mg/dL] (*P* value = 0.04). Also, the FBS level was significantly higher in* H. pylori* seropositive female cases, compared with the seronegative ones (*P* value = 0.04); the current study results were consistent with those of other studies [23]. It seems that the bacteria induce insulin resistance through stimulation of systemic inflammation in patients with* H. pylori *infection; it can be considered as a mechanism in which* H. pylori* induces its atherogenic effects [24].

However, the mechanism in which accordingly* H. pylori* infection causes the increase in lipid profile is not identified completely. Results of an in vitro study (1992) showed that* H. pylori *can increase the absorption of cholesterol from serum and egg yolk; hence, it can be concluded that cholesterol binding to* H. pylori* can reduce absorption of dietary cholesterol [25].

Results of an experimental study showed that interleukin- (IL-) 8 is produced in the* H. pylori* infected mucosa more than normal range [26]. Production of IL-8 in* H. pylori* infection results in the stimulation of mucosa by oxidized LDL and monocytes, and then increases the immigration of lymphocytes-T to smooth muscle cells, and consequently leads to the production of plaque thrombosis [27].

Interleukin-10 (IL-10) is produced by mononuclear cells after the incidence of inflammation. HDL-cholesterol can regulate the production of cytokines by itself. Inflammation, by changing the level of HDL-cholesterol, stimulates the production of IL-10 by circulating mononuclear cells [28].

Some studies showed the positive effect of* H. pylori* eradication therapy on lipid profile; for example, successful eradication of* H. pylori* can reduce the risk of high LDL and low HDL [22].

The* H. pylori* eradication therapy increases apo A and HDL-cholesterol, while total cholesterol and LDL are not changed [28, 29]. A randomized clinical trial evaluated the long-term effect of garlic and nutritional supplements, with a 2-week antibiotic therapy regimen on the treatment of* h. pylori* infection, and lipoproteins and cholesterol profiles [30].

Briefly, at least some patients with* H. pylori* infection showed permanent or long-term complications of atherogenic lipid profile, which can accelerate the incidence of atherogenesis and many other complicated clinical diseases such as coronary heart disease, brain stroke, and peripheral artery occlusive disease [31].

## 5. Conclusion

According to the results of the current study,* H. pylori* can increase the level of LDL and FBS in seropositive male patients; the bacteria also play a role in the incidence of coronary artery disease through affecting atherogenic lipid profile and blood sugar level; but, in seropositive female patients, considering the lower levels of TG, it seems that the atherogenic effect of* H. pylori* mostly affects the level of blood sugar.

## Figures and Tables

**Table 1:** Demographic and Clinical Data of Female and Male Cases.

Variable	Male	Female	*P* value
Age (year)	42.5 ± 3	45 ± 4	0.002
FBS (mg/dL)	105 ± 3	106 ± 2	0.6
TG (mg/dL)	161 ± 3.5	135 ± 4	0.001
Cholesterol (mg/dL)	191.5 ± 10	191.8 ± 8	0.9
LDL (mg/dL)	111 ± 3	112 ± 4	0.89
HDL (mg/dL)	38.3 ± 2	43.7 ± 3	0.001
*H. pylori* seropositive (%)	68	65	0.8

FBS, fasting blood sugar; TG, triglyceride; LDL, low density lipoprotein; HDL, high density lipoprotein.

**Table 2:** Results of *Helicobacter pylori* Seropositive and Seronegative Male Cases.

Variable	*H. pylori* Seropositive Cases	H. pylori Seronegative Cases	*P* value
Age (year)	45 ± 3	40 ± 2	0.001
FBS (mg/dL)	105 ± 8	101 ± 4	0.04
TG (mg/dL)	171 ± 9	168 ± 6	0.7
Cholesterol (mg/dL)	192 ± 11	187 ± 9	0.07
LDL (mg/dL)	113 ± 4	109 ± 6	0.03
HDL (mg/dL)	38.5 ± 3	38.7 ± 4	0.7

FBS, fasting blood sugar; TG, triglyceride; LDL, low density lipoprotein; HDL, high density lipoprotein.

**Table 3:** Results of *Helicobacter pylori* in Seropositive and Seronegative Female Cases.

Variable	*H. pylori* Seropositive Cases	H. pylori Seronegative Cases	*P* value
Age (year)	48 ± 6	41 ± 4	0.001
FBS (mg/dL)	106 ± 9	102 ± 5	0.04
TG (mg/dL)	127 ± 6	155 ± 10	0.001
Cholesterol (mg/dL)	187 ± 12	186 ± 9	0.001
LDL (mg/dL)	110 ± 6	115 ± 8	0.06
HDL (mg/dL)	44 ± 4	43 ± 5	0.15

FBS, fasting blood sugar; TG, triglyceride; LDL, low density lipoprotein; HDL, high density lipoprotein.

## Data Availability

The data used to support the findings of this study are available from the corresponding author upon request.
